# Tailored Immune Responses: Novel Effector Helper T Cell Subsets in Protective Immunity

**DOI:** 10.1371/journal.ppat.1003905

**Published:** 2014-02-20

**Authors:** Ervin E. Kara, Iain Comerford, Kevin A. Fenix, Cameron R. Bastow, Carly E. Gregor, Duncan R. McKenzie, Shaun R. McColl

**Affiliations:** School of Molecular & Biomedical Science, The University of Adelaide, Adelaide, South Australia, Australia; University of Alberta, Canada

## Abstract

Differentiation of naïve CD4^+^ cells into functionally distinct effector helper T cell subsets, characterised by distinct “cytokine signatures,” is a cardinal strategy employed by the mammalian immune system to efficiently deal with the rapidly evolving array of pathogenic microorganisms encountered by the host. Since the T_H_1/T_H_2 paradigm was first described by Mosmann and Coffman, research in the field of helper T cell biology has grown exponentially with seven functionally unique subsets having now been described. In this review, recent insights into the molecular mechanisms that govern differentiation and function of effector helper T cell subsets will be discussed in the context of microbial infections, with a focus on how these different helper T cell subsets orchestrate immune responses tailored to combat the nature of the pathogenic threat encountered.

## Introduction

Bidirectional intercellular communication between innate and adaptive immune systems is crucial for success of immunity to microbial infection. The activation and fate of clonally selected cells of the adaptive immune system is strongly influenced by innate effector cells, and orchestration of adaptive responses to pathogenic microorganisms requires synergistic collaboration with the innate immune system to efficiently resolve infection. Via production of diverse pleiotropic cytokines, effector CD4^+^ T helper (T_H_) cells function to direct efficient immune reactions by dictating the actions of both innate and adaptive arms of the immune system. Through their ability to coordinate innate/adaptive effector cell activity, T_H_ cells directly and/or indirectly influence almost every aspect of an immune response: they provide signals to help B cells undergo class switch recombination (CSR), affinity maturation and differentiation, perpetuate CD8^+^ T cell responses, regulate the recruitment and function of innate effector cells, and contract responses to resolve and/or adjust the magnitude of inflammation.

**Figure 1:**
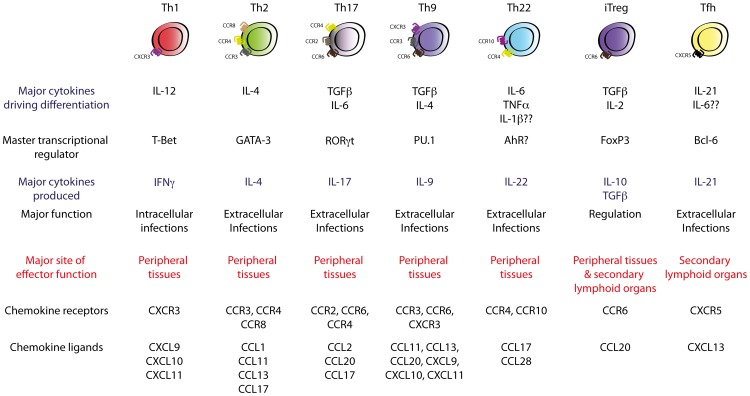
Currently known T_H_ cell subsets. Polarising cytokines encountered during T_H_ cell differentiation drive the expression of subset-specific transcription factors, which imprint subset-specific transcriptomes in the T_H_ cell. These transcription factors define the effector function and migratory capability of the T_H_ cell via regulation of subset-specific cytokines and chemokine receptors.

Pathogen-specific CD4^+^ T cells coordinate immune responses by differentiating into discrete subsets of effector T_H_ cells defined by production of distinct cytokine “signatures”. The specific differentiated state of effector T_H_ subsets is attributed to their expression of subset-specific transcription factors that programme subset-specific transcriptomes, whilst concomitantly suppressing alternative fates the precursor could have assumed [1]. Induction of these transcriptional programmes is predominantly determined by innate-immune-derived cytokines present during MHC-II-restricted T cell receptor (TCR)-mediated activation released into the “immunological synapse” by antigen-presenting cells, particularly by DCs (examples shown in Figure 1). DCs are themselves instructed to produce cytokines following detection of specific pathogen-associated molecular patterns (PAMPs) on foreign microbes through pattern recognition receptors (PRRs) during pathogen encounter in the periphery [2]. Thus, important information regarding the nature of the specific pathogens can be conveyed to developing effector helper T cells that subsequently differentiate into an effector programme equipped with a particular cytokine-secreting repertoire, thereby eliciting a pathogen-tailored immune response.

These views of helper T cell differentiation and function were first introduced by Mosmann and Coffman in 1986, who demonstrated that T cell clones were divisible into two subsets, termed T_H_1 and T_H_2, based on their mutually exclusive production of interferon (IFN)-γ or interleukin (IL)-4, -5, and -13, respectively [3]. This subdivision was of major significance as IFN-γ-producing T_H_1 cells were subsequently shown to be critical in host defences against intracellular pathogens by activating cell-mediated immunity, whilst T_H_2-driven responses were essential for efficient humoral responses against extracellular microbes. The T_H_1/T_H_2 paradigm served as a useful conceptual construct for understanding how T_H_ cells controlled different arms of the immune system, and dysregulation of T_H_1/T_H_2 responses has since been implicated in the pathogenesis of many immune-related disorders such as autoimmune and allergic disease. Development of techniques such as multi-parameter flow cytometry and engineering of fate-mapping cytokine reporter mice has recently facilitated major progress in T_H_ cell biology, with seven functionally unique T_H_ subsets now described. These comprise T_H_1, T_H_2, T_H_17, follicular helper T cells (T_FH_), inducible T regulatory cells (iTreg), and the most recently described and least well-characterised subsets, T_H_9 and T_H_22 cells, each of which is produced upon antigen presentation in the presence of specific cytokines or sets of cytokines (Figure 1). In this review, recent insights into the mechanisms that govern differentiation, migration, and function of effector T_H_ cells will be discussed in the context of microbial infection, focussing on the contribution of emerging subsets of effector helper T cells, with less emphasis on T_H_1 and T_H_2 subsets, whose function has been well-established and is described elsewhere [4]. The function of Tregs in protective immunity will also not be discussed in this review as this has been the subject of recent comprehensive review elsewhere [5].

## T Helper 1 (T_H_1) and T Helper 2 (T_H_2)

T_H_1 differentiation from naïve precursors is initiated by signal transducer and activator of transcription (STAT)-1 activation downstream of type 1 interferon, IFN-γ and IL-27 signalling, which induces expression of the T_H_1-specific master transcription factor T-bet [6]–[9]. This process enables activated CD4^+^ T cell responsiveness to DC-derived IL-12 via T-bet-mediated induction of the high-affinity IL-12 receptor beta 2 chain on the cell surface [10]–[12]. IL-12 signalling through STAT4, together with T-bet, directly transactivates the *Ifng* gene, which further promotes T_H_1 differentiation via STAT1 activation in an autoregulatory feedback loop [13]–[15]. T-bet also drives expression of the chemokine receptor CXCR3, which facilitates T_H_1 migration to inflamed sites of pathogen encounter where CXCL9, CXCL10, and/or CXCL11 are produced (Figure 1) [12], [15]–[17]. T_H_1 cells orchestrate the cell-mediated cytotoxic response against intracellular pathogens principally via provision of IFNγ to enhance macrophage activation and promote activation of antigen-specific cytotoxic T lymphocytes (CTLs). Classical infections controlled or cleared by effective T_H_1 responses include intracellular bacteria such as *Listeria monocytogenes*, *Salmonella* species, and *Mycobacterium tuberculosis*, intracellular parasites such as *Leishmania donovani*, and a number of viral pathogens [18]–[25]. In addition, T_H_1-derived production of the pro-inflammatory cytokine IL-21 has also been shown to be a key regulator of the long-term maintenance and functionality of antigen-specific CTLs important for protection against both acute and chronic infection with lymphocytic choriomeningitis virus (LCMV) [26]–[28].

Despite the appreciation of the existence of the T_H_2 subset for more than 25 years, the molecular mechanisms that govern T_H_2 differentiation remain controversial. Early reports demonstrated that the T_H_2 differentiation programme is set up via STAT6 activation downstream of IL-4 signalling, directly transactivating the T_H_2-lineage-specific transcription factor GATA-3 that in turn induces expression of the T_H_2-specific cytokine genes *Il4*, *Il5*, and *Il13* [29]–[36]. However, recent studies suggest that T_H_2 cell induction may be far more complex than originally described, with reports that T_H_2 differentiation can occur independently of the STAT6/IL-4 axis [37] and may require additional cytokines including IL-2, IL-25, IL-33, and thymic stromal lymphopoietin (TSLP) (reviewed in [4], [38]). Nevertheless, production of IL-4, -5, and -13 by T_H_2 cells plays important roles in the activation and recruitment of basophils, induction of eosinophilia, regulation of antibody-dependent cell-mediated cytotoxicity (ADCC) mechanisms, and, by acting on resident cells at sites of inflammation, creates a hostile environment that favours extracellular microbial expulsion [4]. T_H_2 cells express the chemokine receptors CCR3, CCR4, and CCR8, and thus migrate to sites expressing their ligands in response to infection (Figure 1) [39], [40]. Effective T_H_2 responses are required for host defence against extracellular parasites such as *Schistosoma mansoni* or *Trichuris muris* [41]–[44].

The subdivision of T cells into T_H_1 and T_H_2 subsets has utility in understanding how the adaptive immune system tailors responsiveness to different types of pathogens by directing the activation of distinct immune components. Clearly however, the T_H_1 and T_H_2 subdivision is an oversimplification as the substantial pathogen diversity warrants more than two broad types of immune response. Therefore, it is perhaps surprising that it was only relatively recently that the T_H_1 and T_H_2 paradigm has been expanded to definitively include additional subsets of T cells and the role of these subsets in responses to distinct microbial challenges has been interrogated. In the remainder of this review, these emerging subsets of T cells and their role in protective immunity will be described.

## T Helper 17 (T_H_17)

It was not until 2005 that a third major population of effector T_H_ cells was described on the basis of the observation that peripheral CD4^+^ T cells could differentiate into a distinct lineage in a GATA-3– and T-bet-independent fashion [45], [46]. Early reports suggested that these cells did not produce molecules commonly associated with T_H_1 or T_H_2 subsets but characteristically expressed the highly pro-inflammatory cytokines IL-17A and IL-17F, and were subsequently designated T_H_17. However, later studies have demonstrated that subsets of T_H_17 cells can produce IFN-γ, IL-4, or IL-13 under certain circumstances [47]–[50], although the function of T_H_17-derived IFN-γ, IL-4, or IL-13 has yet to be explored in infectious models. T_H_17 differentiation requires IL-6 and is also promoted by the otherwise immunosuppressive cytokine TGF-β1 (Figure 1) [51]–[53]. Signal transduction downstream of these cytokines, including STAT3 activation downstream of the IL-6 receptor, induces expression of the T_H_17-lineage-specific transcription factor RORγt, which directly transcribes the T_H_17-lineage-specific cytokines *Il17a* and *Il17f* (Figure 1) [54]–[57]. IL-6-mediated induction of IL-21 during T_H_17 cell differentiation is reported to reinforce T_H_17-lineage commitment via STAT3 activation downstream of the IL-21 receptor in an autocrine manner [53], [58], [59]. It has also been reported that autocrine TGF-β1 promotes T_H_17 cell differentiation *in vivo* [60]. Whilst addition of IL-6 and TGF-β1 into naïve CD4^+^ T cell cultures does indeed drive T_H_17 cell differentiation, the *in vivo* requirements of T_H_17 cell differentiation are far more complex than these *in vitro* conditions. Recent reports suggest that T_H_17 cell differentiation can be induced independent of TGF-β1 signalling when driven by the inflammatory cytokines IL-6, IL-1β, and IL-23 [61]. T_H_17 cells induced independently of TGF-β1 (termed T_H_17(23), owing to their requirement for IL-23) appear to possess more inflammatory characteristics than conventional TGF-β1-driven T_H_17 cells (T_H_17(β)). Furthermore, it has also recently been shown that IL-6 and TGF-β3 drive differentiation of T_H_17 cells that are functionally and molecularly distinct to the conventional T_H_17(β) cell [62]. Thus, it is likely that T_H_17 cells in any given response may comprise a heterogeneous population of distinct types of T_H_17 cells that arise in discrete cytokine microenvironments, possess distinct but similar transcriptomes, and subsequently possess different cytokine-secreting repertoires and functions. However, these hypotheses remain to be extensively tested. Characteristically, T_H_17 cells express the chemokine receptor CCR6 and their homing is thereby regulated by the CCR6 ligand, CCL20, at sites of infection [63]. The near-ubiquitous expression of the IL-17 receptor on non-haematopoietic cells facilitates the broad physiological functions of T_H_17 cells during inflammation. Through the induction of the inflammatory chemotactic factors CXCL1, CXCL2, CXCL5, and CXCL8 at sites of inflammation via production of IL-17A/F, IL-22, and GM-CSF, T_H_17-mediated responses are dominated by the inflammatory and phagocytic functions of neutrophils [64]. Other T_H_17-mediated functions include induction of antimicrobial peptides (including S100 proteins and β-defensins), promotion of granulopoiesis via induction of G-CSF, and enhancement of monocyte and neutrophil activation to promote their phagocytic activity [64].

Whilst T_H_17 cells represent, in most cases, the major source of adaptive IL-17 during microbial infection, IL-17 elicited from non-T_H_ cell sources can also be a determining factor in host defence. Invariant natural killer T (iNKT) cells, natural killer (NK) cells, γδ-T cells, and type 3 innate lymphoid cells (ILC) (including lymphoid tissue-inducer (LTi) cells) have all been shown to produce protective innate-derived IL-17 in response to infection [65]–[68]. The importance of IL-17 derived from non-T_H_17 cell origins has recently been reviewed elsewhere [69], [70].

**Figure 2:**
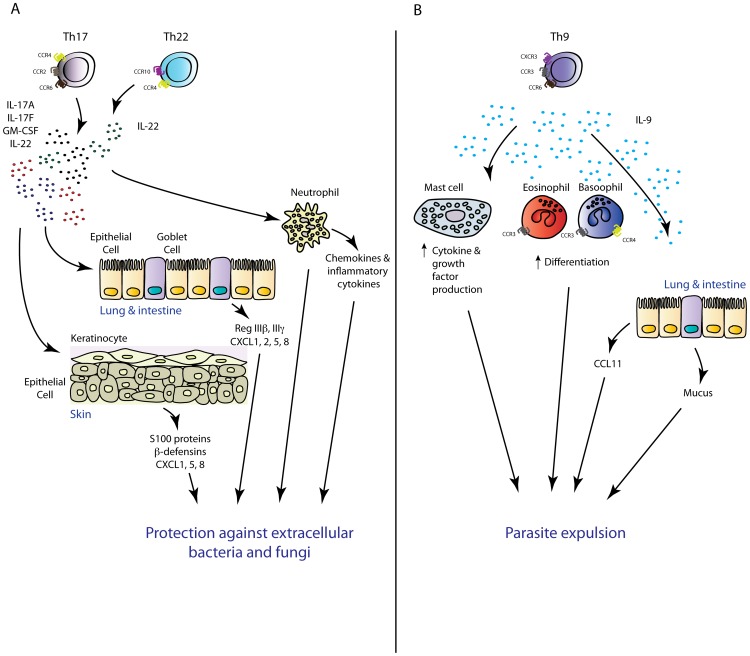
Novel T_H_ subsets in inflammation. (**A**) T_H_17 and T_H_22 cells have overlapping functions in the mouse. Via production of the inflammatory mediators IL-17A, IL-17F, GMCSF (T_H_17), and IL-22 (T_H_22), these T_H_ subsets mediate protective immunity against extracellular pathogens intimately associated with mucosal barriers. (**B**) T_H_9-cell-derived IL-9 may play an important role in antiparasitic immunity via mediating mast cell activation and mastocytosis, increasing the chemotactic potential of an inflammatory site via regulation of inflammatory chemokine production, and promote basophil and eosinophil function.

Recent gene-knockout studies have demonstrated the vital importance of IL-17-mediated inflammatory responses for host defences at epithelial barriers, particularly against notoriously persistent extracellular bacteria and fungi (Figure 2, panel A). Seminal work by Ye et al. demonstrated the critical importance of IL-17 for protective immunity in a murine model of extracellular bacterial infection using *Klebsiella pneumoniae* in IL-17R-deficient mice [71]. These mice were more susceptible to intranasal *K. pneumoniae* infection relative to WT counterparts, which correlated with significant delay in neutrophil recruitment into the alveolar space and heightened dissemination of bacteria into the circulation [71]. Numerous studies that followed confirmed the essential role of IL-17 in host protection against *K. pneumoniae* [71]–[73]. Mice infected with other extracellular bacteria including *Citrobacter rodentium* [74], [75], *Bordetella pertussis* [76], [77], *Porphyromonas gingivalis* [78], [79], or *Streptococcus pneumoniae* [80], [81], for example, also mount protective IL-17 responses, and disruption of IL-17A or its receptor leads to exacerbated bacterial burden or dissemination, increased disease susceptibility resulting from defective induction of CXC chemokines, and impaired neutrophil recruitment to sites of bacterial inoculation. Mice with deficiencies in IL-23, a cytokine axis critical for the stabilisation of the T_H_17 phenotype [82], also display exacerbated pathology associated with numerous extracellular bacterial infections. IL-23p19-deficient mice, akin to IL-17– or IL-17R-deficient mice, also fail to effectively mount protective IL-17 responses to *C. rodentium* [52] and *K. pneumoniae* [72] infections. In the absence of these components of the T_H_17– and IL-17-producing innate cell response, bacterial clearance is impeded, leading to augmented bacterial dissemination and disease susceptibility associated with reduced early IL-17-mediated neutrophil infiltration. Importantly, administration of recombinant IL-17 into IL-23-deficient infected mice restored neutrophilia at sites of inoculation [72], demonstrating the critical importance of the IL-23/IL-17 axis in host defence against various extracellular bacterial infections. Whilst these studies strongly implicate a protective role for the IL-23/IL-17 axis in protection against extracellular infections at epithelial surfaces, the precise cellular origin of IL-17 remains controversial. In the context of *C. rodentium* infection, both an early innate and late adaptive source of IL-17 is thought to be crucial to host protection. Interestingly, IL-17 responses early during *C. rodentium* infection were shown to be elicited from a specialised subset of CD4^+^ T cells present within the lamina propria (LP) [83]. Differentiation of these cells was dependent on the innate immune sensor receptors NOD-1 and NOD-2, which were shown to regulate intestinal DC-derived IL-6 and subsequent differentiation of these LP-resident “early” T_H_17 cells. Importantly, NOD-1/NOD-2 deficiency did not alter IL-17A production during the late “adaptive” phase of infection suggesting that these sensors specifically regulate early CD4^+^-T-cell-derived IL-17. Thus, based on their rapid induction and distinct dependency on NOD-1/NOD-2, these early T_H_17 cells were termed innate T_H_17 (iT_H_17) cells. iT_H_17-derived early IL-17 is not restricted to *C. rodentium* infection as the same study demonstrated that these cells also contribute to defence against *S. typhimurium*, another attaching and effacing bacterium [83]. Other cells, including γδ-T and ILC3 cells, have also been shown to produce IL-17 following extracellular bacterial challenge [83]–[86]. Thus, it will be important to delineate the source of IL-17 in the context of extracellular bacterial infections to fully understand the function of T_H_17 cells in these settings. Experiments where IL-17 is specifically deleted in the T cell compartment will be required to obtain this information.

The importance of IL-17-driven inflammation in the context of antifungal host defence has also been established. In mice and men, pathogen-specific T_H_17 responses have been shown to confer protection against the dimorphic filamentous fungus *Candida albicans* [87]. Within the memory CD4^+^ T cell pool of healthy volunteers, *Candida*-specific T_H_ cells are enriched within the T_H_17 subset and significantly heightened numbers of IL-17-producing cells in peripheral leukocytes of acute *Candida*-infected patients have been documented compared to healthy controls upon restimulation with *Candida* antigens [88]. Moreover, chronic mucocutaneous candidiasis patients have diminished numbers of IL-17A-producing cells within the peripheral leukocyte pool compared with acutely infected patients and healthy controls [89]. These data, and observations that patients with autosomal dominant hyper-IgE syndrome, characterised by defects in T_H_17 differentiation due to mutations in the T_H_17-polarising transcription factor STAT3 [90]–[92], are more susceptible to *Candida* and other fungal infections, support an important role for the T_H_17 response in effective antifungal immunity [93]. More detailed analyses of the functional role of IL-17 in fungal immunity have come from murine models of experimental fungal infection. In line with human studies, mice with a deficiency in IL-17A or its receptor are more susceptible to experimental fungal infection. A role for the IL-17 axis in antifungal immunity in mice was first described in 2004, in a study in which intravenous infection of IL-17R-deficient mice with *Candida* led to decreased survival rates and augmented fungal burden in the kidney [94]. In a model of oropharyngeal candidiasis (known as “thrush”), mice with deletions in IL-23p19, IL-17RA, or IL-17RC developed exacerbated thrush lesions associated with augmented fungal burdens, whilst mice deficient in T_H_1 effector cytokines IFN-γ or TNF-α were resistant to oral infection [95]–[97]. Critical requirements for the IL-23/IL-17 axis in protective immunity have also been described in murine models of dermal candidiasis [98]. Collectively, these studies demonstrate that the IL-17 response is essential for protective immunity against disseminated, skin, or mucosal *Candida* infection. Whilst it was believed that T_H_17 cells represent the major cellular source of protective IL-17 against *Candida*, a recent study demonstrated that ILC3-derived IL-17 was the critical source of this cytokine in an oropharyngeal candidiasis infection model [67]. Immunity to oropharyngeal infection with *Candida* was not altered in Rag-deficient or T-cell-deficient animals, suggesting that T_H_17-cell-derived IL-17 was not an important component of host defence. Antibody-mediated depletion of all ILCs, as well as deletion of ILC3 cells using Rorc-deficient mice, led to enhanced susceptibility to *Candida* infection, implicating ILC3 cells as the crucial cellular source of protective IL-17 in this model. Further investigation is required to determine the contribution of innate and adaptive sources of IL-17 in other models of primary *Candida* infection or other fungal pathogen models where IL-17 has been shown to confer protection including *Cryptococcus neoformans* [99], *Aspergillus fumigatus* [100], and *Pneumocystis carinii* [101]. Taken together, recent data have suggested that both T_H_17– and innate-cell-derived IL-17 play important roles in the context of extracellular bacterial and fungal infections. It is likely that innate IL-17 is crucial as the first line of defence whilst the pathogen-specific T_H_17 cell response plays more prominent roles during the late phase of infection and in recall challenges. In support, T_H_17 cell recall responses are required for effective clearance of *Candida* and *K. pneumoniae* infection [102], [103]. T_H_17 responses have also been shown to be required for vaccine-induced protection to the endemic fungal pathogens *Coccidioides posadasii*, *Histoplasma capsulatum*, and *Blastomyces dermatitidis* [104] as well as a number of other mucosa-associated pathogens [105].

As outlined above, host defences against intracellular pathogens are classically considered to be coordinated by the T_H_1 domain. However, recent data have also revealed a potential role for T_H_17 cells in the context of intracellular microbial infection [106]. Pulmonary infection of mice with the intracellular bacterium *Francisella tularensis* induced a protective T_H_17 response [107], [108], and deletion of the IL-23/IL-17A axis, but not IL-17F or IL-22, increased susceptibility to pulmonary tularemia [108]. Interestingly, the reported biological function of IL-17A in this model was induction of IL-12 and IFN-γ production from APCs, subsequently promoting antigen-specific T_H_1 responses [108]. The ability of IL-17A to regulate T_H_1 responses in the context of microbial infections is not limited to the tularemia model, with reports that IL-17 can influence adaptive immunity to pulmonary *Chlamydia muridarum* [109] and *Mycobacterium bovis* BCG [110] infection via similar mechanisms. However, impaired T_H_17-mediated neutrophil recruitment is also likely to contribute to these observed phenotypes [108]. Indeed, IL-23/T_H_17-dependent neutrophil responses are important components of protective immunity to other intracellular bacteria infections such as *Mycoplasma pneumonia* [111] and *Salmonella enterica* serotype Typhimurium [112], [113]. Taken together, these studies suggest that T_H_17 cells function in concert with T_H_1 cells to efficiently resolve some intracellular bacterial infections. The molecular and cellular basis of how these pathogens elicit T_H_17 cell responses despite induction of a priming environment dominated by T_H_1-polarising cytokines that antagonize T_H_17 differentiation remains to be determined. Owing to inherent differences in PRR stimulation by various bacteria, it is possible that certain bacteria effectively induce potent IL-12/IFN-γ responses, whilst other bacterial pathogens require an additional IL-17-dependent mechanism for host IL-12/IFN-γ production to resolve intracellular infection. Moreover, the requirement of neutrophil responses to intracellular infection poses something of a paradox, as these cells are not thought to elicit robust effector responses against intracellular pathogens. However, it may be that T_H_17-driven neutrophil responses in these settings are active against the extracellular phase of a pathogen's life cycle, i.e., *trans*-epithelial bacterial entry.

Virus-specific IL-17-producing CD4^+^ T cells have also been detected in mice following herpes simplex virus (HSV) [114], Theiler's murine encephalomyelitis virus (TEMV) [115], and vaccinia virus (VV) [116] infection, among others [20], albeit at a lower magnitude than the prototypical antiviral T_H_1 response. In these settings, T_H_17 responses appear to be detrimental to the host as IL-17R-deficiency, or neutralisation of IL-17A, reduced HSV-induced stromal keratitis [114]. Furthermore, neutralisation of IL-17 during chronic TEMV infection increased viral clearance and enhanced cytotoxic T cell responses [115], and neutralisation of IL-17 during VV infection decreased the size of primary and satellite lesions, and promoted viral clearance [116]. Nevertheless, protective roles have also been ascribed to T_H_17-derived cytokines in some viral infections [117]. This precarious balance of protective versus harmful effector functions of T_H_17 cells has also been observed in the context of parasitic infections where IL-17 has been shown to promote host defence against intestinal *Toxoplasma gondii* infection at the expense of heightened immunopathology [118] and contribute to control of *S. mansoni* infection in the lung [119], yet enhance immunopathology associated with schistosomiasis when mice are infected with *S. mansoni* in the liver [120], [121].

Thus, it is apparent that the T_H_17 subset is predominantly associated with host defence against extracellular pathogens via IL-17-mediated mobilisation of neutrophil responses. T_H_17 cells may also serve as an important adjunct to T_H_1 responses in certain intracellular infections by inducing neutrophil responses that may function during the extracellular life cycle of intracellular pathogens, or by influencing APCs to promote T_H_1 polarisation in some intracellular bacterial infections that fail to induce efficient pathogen-specific T_H_1 cell differentiation. Conversely, the T_H_17 response can be detrimental to the host in that it can contribute to viral persistence and promote pathological inflammation associated with parasitic infection, which thereby presents these cells as a potential therapeutic target to limit pathology in these settings. Given that optimal immunity to extracellular infections is highly dependent on the function of antibodies, the critical importance of generating pathogen-specific memory T_H_17 cells in vaccine development under conditions where a primary live infection elicits protective T_H_17 cell responses is often overlooked. Further study of this system will facilitate design of vaccines that result in improved memory T_H_17 cell development in synergy with robust antibody responses such that both humoral and cell-mediated arms of the immune system enter into immunological memory. Such strategies are indeed currently under development as is evident by promising vaccine candidates that elicit pathogen-specific T_H_17 cells in the context of anti–*S. pneumoniae* immunity [122].

## T Helper 22 (T_H_22)

Recently, a subset of human T_H_ cells dedicated to production of the cytokine IL-22 has been described and proposed to be a separate lineage of T_H_ cell, designated T_H_22 [123], [124]. IL-22 is a pro-inflammatory member of the IL-10 family of cytokines that appears to be particularly important for driving inflammatory responses at cutaneous and mucosal surfaces [125]. T_H_22 differentiation from naïve precursors has been reported to be IL-6– and TNF-α-dependent [123] (Figure 1) and studies suggest that IL-22 production by these cells is transcriptionally regulated by the ligand-activated transcription factor aryl-hydrocarbon receptor (AhR) [123], [124], [126], [127] (Figure 1). Expression of the IL-22 receptor is restricted to stromal cells of the skin, intestine, liver, kidney, pancreas, and lung [128], [129], implicating T_H_22 cells as an important mediator of the interaction of the immune system with the non-hematopoietic environment. T_H_22 cells have been shown to express the chemokine receptors CCR4, CCR6, and CCR10 [123], [124], the ligands of which are known to regulate homing to these organs.

In mice, IL-22 elicited from T_H_ cells appears to be restricted to the T_H_17 cell subset [130], with very few studies having detected *bona fide* IL-17A^−^ T_H_22 cells *in vivo*. For this reason, evaluating the function of this potential novel human T cell subset using murine models of microbial infections currently presents significant challenges. The T_H_22 response clearly shares many similar features with the T_H_17 response, as evidenced by the lack of *obvious* divergence between these responses in mice, and the common reliance on AhR signalling for aspects of their function [126]. Akin to the function of other T_H_17-derived cytokines, IL-22 ligation with its receptor markedly induces expression of multiple antimicrobial peptides including the S100 proteins S100A7–A9, β-defensins, the intestinal antimicrobial peptides RegIII-β and -γ, and stimulates production of protective mucus elicited from goblet cells (Figure 2, panel A) [129]–[132]. IL-22 also upregulates expression of the inflammatory chemokines CXCL1, CXCL2, and CXCL5, which act in synergy with IL-17 to induce a chemotactic environment that promotes neutrophilia at sites of infection [133]. In addition to its antimicrobial and pro-inflammatory effects, IL-22 also plays an important role in tissue regeneration and wound healing by promoting epithelial cell proliferation and inducing expression of anti-apoptotic proteins [134].

IL-22 appears to play a dichotomous role in host defence depending on the nature of the pathogen and site of infection. Protective functions of IL-22 have been described in the context of extracellular pathogen infection of the lung and intestine including *K. pneumoniae* and *C. rodentium*. In most cases, IL-22 was essential for control of bacterial replication and dissemination, most likely in part due to the ability of this cytokine to potently induce antimicrobial peptide production by epithelial cells at these barrier surfaces [132], [133]. In line with the protective role for T_H_17 responses in antifungal immunity, IL-22-producing CD4^+^ T cells have also been detected within the *Candida*-specific memory T cell pool of healthy patients [135], and are defective in patients with chronic mucocutaneous candidiasis [89], [136], [137]. However, the function of IL-22 in experimental *C. albicans* infection remains controversial [98], [138]. In a murine model of oropharyngeal candidiasis, host defence was predominantly mediated by IL-17, not IL-22 [96], whilst protective immunity to *C. albicans* infected intragastrically was dependent on IL-22-mediated production of antimicrobial peptides including S100A8 and S100A9, which prevented yeast dissemination to the kidneys and stomach [139].

Numerous studies have suggested that immunity to intracellular pathogens or parasites does not appear to rely on IL-22. Host defence against the intracellular pathogens *Mycobacterium avium* and *L. monocytogenes*, or the parasitic pathogen *S. mansoni*, is IL-22-independent [140]–[142]. Unlike *K. pneumoniae* and *C. rodentium*, these pathogens are not intimately associated with mucosal or cutaneous barriers, which may underlie the redundant role of IL-22 in these settings. Furthermore, IL-22 has been shown to be detrimental in a murine model of oral *T. gondii* infection [140], [143]. In this model, IL-23 promoted development of ileitis in an IL-22-dependent manner. Whilst no difference in protozoan burden was documented between WT and IL-22-deficient mice, WT mice succumbed to infection due to intestinal necrosis, whereas IL-22-deficient mice displayed increased survival rates with only minor inflammation evident. Flow cytometric analyses implicated CD4^+^ T cells in the lamina propria as the major source of IL-22, which contributed to *T. gondii*–induced panileitis principally via an immune response directed against gut microbiota rather than the protozoan pathogen. These data suggest that particular microbial agents can induce detrimental IL-22-mediated pathogenic inflammation [143]. On the contrary, studies have implicated protective roles for IL-22 in certain intracellular pathogen infections including experimental influenza and dengue infectious models [144]–[146]. Despite reports that IL-22 deficiency or neutralisation does not alter the outcome of *M. tuberculosis* infection in mice [140], [147], Scriba et al. demonstrated that a substantial proportion of mycobacteria-specific T_H_ cells from healthy *M. tuberculosis*–exposed individuals produce IL-22 and are distinct from T_H_17 and T_H_1 cells, implicating IL-22 as an important cytokine axis in human anti-mycobacterial immunity [148]. These reported differences between mice and men in *M. tuberculosis* infection support the notion that CD4^+^-T-cell-derived IL-22 plays a more prominent role in the human than murine immune system, at least under certain circumstances.

Despite the fact that T_H_22 cells are not clearly distinguishable from the T_H_17 subset in mice, experiments to specifically evaluate the significance of T-cell-derived IL-22 in models of microbial infection have been performed. T_H_22 cells have been detected in experimental coxsackievirus-B3-induced myocarditis where they appear to exacerbate acute viral-induced myocarditis associated with increased cardiac viral replication, heightened cardiomyopathy, and reduced survival rates [149]. More recently, a study by Basu et al. demonstrated, for the first time, the function and protective efficacy of T_H_22-derived IL-22 in the context of microbial infection [150]. Significant expansion of IL-22-producing CD4^+^ T_H_ cells that lacked expression of IL-17A occurred in the colonic lamina propria during the late phase of *C. rodentium* infection, implicating T_H_22 cells as the predominate T_H_ subset mediating host protection to this enteropathogenic bacterium. Infection of IL-6-deficient mice led to profound defects in lamina-propria-resident T_H_22 cell numbers, but not IL-22 production from other cells, relative to IL-23-deficient mice or WT counterparts, illustrating the importance of IL-6 in regulating T_H_22 differentiation. The importance of T-cell-derived IL-22 in protective immunity to this pathogen was reflected in the marked decline in survival of mice treated with neutralising antibodies to IL-22 administered after the peak of the innate immune response. Moreover, adoptive transfer of *in vitro*–generated T_H_22 cells, but not *in vitro*–generated T_H_17 cells, into *Il22* ^−/−^ mice rescued recipient mice from pathogenic inflammation [150]. These experiments are the first to definitively demonstrate the existence and function of T_H_22 cells during enteropathogenic bacterial infection. Further detailed studies are required to explore the function of T_H_-cell-derived IL-22 in other infectious models.

It is important to appreciate that, similar to other T_H_-cell-derived cytokines, IL-22 production is not restricted to the CD4^+^ T cell compartment. Various other cells, including γδ-T, NKT, and CD8^+^, have the ability to produce IL-22 that participates in host defence against microbes [70], [132], [139], [142]. More specifically, ILC3s have been shown to be a dominant innate source of IL-22 during infection [125]. Thus, in order to delineate the function of T_H_22-derived IL-22 in the context of microbial infection, mice with T-cell-specific deletions of IL-22 will be required. However, to our knowledge, these reagents have yet to be developed.

Given the recent discovery of the T_H_22 subset, limited studies have been carried out to date regarding the function of T_H_22 cells in host defence to microbes. As discussed above, current data suggest that the T_H_22 subset, in most cases, has overlapping functions with the T_H_17 lineage in mice, in contrast to the human system where IL-22-secreting T cells potentially form a distinct lineage. It is now important to dissect how and why certain infections elicit IL-22 responses that are favoured over IL-17-mediated immunity in humans. The results of such studies may provide crucial insights into how the balance of T_H_22/T_H_17 cells defends against certain pathogens and may lead to the development of vaccines tailored to particular microbial threats.

## T Helper 9 (T_H_9)

IL-9 represents one of the most understudied cytokines in the field of T_H_ cell biology despite its diverse biological effects on numerous cell types of myeloid, lymphoid, and stromal origin [151]. IL-9 was first associated with T_H_2-mediated responses following reports that IL-9 expression in T cells was high in T_H_2-prone BALB/c mice relative to the T_H_1-prone C57Bl/6 mouse strain during the course of *L. major* infection [152]. Subsequent studies implicated a protective role for IL-9 in T_H_2-driven responses during murine parasitic infections [153], [154], with IL-9 levels in mesenteric lymph nodes correlating with expansion of T_H_2 cell populations and a requirement of IL-9 for CSR to the “type-2” antibody isotypes IgG_1_ and IgE [155], [156]. Furthermore, an *in vivo* requirement for IL-4, a crucial mediator of T_H_2 differentiation, for induction of IL-9 expression by T cells was later demonstrated in *L. major*–infected BALB/c mice [152]. The results of these studies led to the classification of IL-9 as a T_H_2-derived pro-inflammatory cytokine. However, despite the clear association between IL-9 and T_H_2 responses, recent reports of high-level IL-9 production in macrophage– and neutrophil-dominated inflammatory settings were counter to previous conceptions that IL-9 was elicited from T_H_2 cells [157]. These findings have recently been reconciled with the discovery that naïve T cell priming in the presence of IL-4 and TGF-β drives differentiation of a functionally disparate subset of IL-9-secreting T_H_ cells, designated T_H_9 (Figure 1) [158], [159]. Subsequent studies have called into question the requirement of IL-4 in T_H_9 differentiation with reports that IL-4R signalling induces expression of suppressor of cytokine signalling (SOCS) family member cytokine-induced SH-2 protein (CIS), which inhibits STAT5/STAT6 signalling and subsequent T_H_9 cell differentiation [160]. Indeed, IL-9 production in T cells has been shown to be independent of IL-4 when activated in the presence of TGF-β1 and IL-1α [161]. *In vivo*, the molecular requirements for T_H_9 cell induction may involve many additional stimuli including IL-25, TSLP, 1,25-dihydroxyvitamin D3, programmed cell death ligand (PD-L) 2, cyclooxygenase (COX)-2, and tumor necrosis factor receptor superfamily member 4 (OX40) [162]. Furthermore, data suggesting that the T_H_9 programme is unstable and highly prone to plasticity have raised questions as to whether this IL-9-secreting CD4^+^ T cell indeed represents a distinct differentiation lineage. These aspects of T_H_9 cell biology will not be discussed in this review but have been recently reviewed elsewhere [162], [163]. Differentiation of this subset is thought to require the transcription factors PU.1, IRF4, and BATF [164]–[166]. We have recently examined chemokine receptor expression by T_H_9 cells and have shown that these cells express a broad range of trafficking receptors, including CCR3, CCR6, and CXCR3 [167]. Notably, these receptors are also characteristically expressed by other T_H_ cell subsets (T_H_2, T_H_17, and T_H_1, respectively) suggesting that T_H_9 cells have the capability of being recruited to, and contributing to multiple, functionally distinct forms of inflammatory lesions. Whilst CCR4 expression by T_H_9 cells generated in our models was not detected, recent work has suggested that these cells also express CCR4 and CCR8, which would presumably allow these cells to traffic to cutaneous sites of inflammation [166].

Given that the description of this new T_H_ cell subset came years after initial studies into the role of IL-9 in the context of microbial infections were carried out, the function of IL-9 in protective immunity will be discussed with conjecture on the role of *bona fide* T_H_9 cells in these settings. Studies using IL-9 transgenic (IL-9^Tg^) mice have emphasized the importance of this cytokine in the control of certain intestinal parasitic infections (summarized in Figure 2, panel B). Following infection with *Trichinella spiralis* or *T. muris*, IL-9^Tg^ mice developed enhanced intestinal mastocytosis and augmented pathogen-specific IgG_1_ responses, which led to rapid parasitic expulsion from the gut [153], [154]. Furthermore, treatment of mice with neutralising antibodies to IL-9 during the course of *T. muris* infection diminished immunity to this pathogen [168]. In line with the protective phenotypes observed in IL-9^Tg^ mice, a specific role for T_H_9-derived IL-9 in protective immunity to intestinal nematode infection was more recently assessed using mice in which TGF-β signalling, a crucial mediator of T_H_9 differentiation, was specifically deleted in the CD4^+^ T cell compartment. Infection of these mice with *T. muris* augmented worm burden and reduced IL-9 but not IL-13 production in mesenteric lymph nodes [159]. In these models, IL-9 appears to predominantly function via activation of mast cells, the inflammatory mediators from which promote seminal processes required for effective parasite expulsion such as induction of eosinophilia, increased intestinal permeability and contractility, and mucus production. Recent work by Licona-Limon et al. using novel IL-9 reporter mice (termed Interleukin Nine Fluorescent Reporter: INFER) and newly generated IL-9-deficient mice on a BALB/c background has revealed a critical and nonredundant role for T_H_9 and IL-9 in host defence to *N. brasiliensis* [169]. GFP reporter activity was detected in CD4^+^ T cells and type-2 ILCs (ILC2: a known prominent source of IL-9 in numerous type-2 models) in both lungs and mediastinal lymph nodes during the course of *N. brasiliensis* infection. IL-9-GFP detection in CD4^+^ T cells in the lung peaked early and declined during the course of infection, whilst IL-9-GFP^+^ ILC2s were detectable early and remained present throughout, suggesting a transient window of CD4^+^-T-cell-derived IL-9 in this model. Adoptive transfer of T_H_9 cells into IL-9-deficient mice led to enhanced worm expulsion, demonstrating that T_H_9-derived IL-9 was an important contributor to IL-9-dependent immunity in this model. The results of this study also elucidated numerous other unknown aspects of T_H_9 cell biology including the demonstration of functional differences between T_H_2 and T_H_9 cells in host protection, despite prior reports concluding that the function of these two subsets in other models substantially overlapped. Surprisingly, transferred T_H_9 cells, but not T_H_2 cells, into infected Rag2-deficient hosts decreased worm burden. T_H_9-mediated protection correlated with increased numbers of mast cells and basophils in lungs and spleens of infected mice, implicating these innate effector cells as the key responding cell types to IL-9-mediated immunity. Moreover, recent findings by Turner et al., using IL-9 fate mapper reporter mice (termed IL-9^Cre^R26R^eYFP^ mice, which permanently label cells with enhanced YFP (eYFP) that have expressed IL-9 irrespective of their current IL-9 expression status) and IL-9R-deficient animals, support the notion that IL-9 plays a critical role in host defence against *N. brasiliensis* infection [170]. Both eYFP^+^ CD4^+^ T cells and ILC2 cells were detected in the lung during infection. IL-9 in this setting was demonstrated to positively regulate IL-5 and IL-13 responses, likely ILC2-derived IL-5 and IL-13 as T_H_2 cell numbers were unchanged in IL-9R-deficient animals, promote ILC2 cell survival, drive lung tissue repair mechanisms, and promote eosinophil recruitment and alternative activation of macrophages. These studies highlight the critical importance of both T_H_9 and ILC2-derived IL-9 in host defence to *N. brasiliensis*. However, the results of these studies are not in keeping with a prior study, which demonstrated that IL-9-deficient mice on a mixed genetic background (129×C57Bl/6 (F_2_)) effectively control infection with *N. brasiliensis* [171]. The conflicting results of these studies warrant further investigation of IL-9 function in anti–*N. brasiliensis* immunity but suggest that the overall importance of IL-9 depends on complex multi-genetic factors. IL-9 deficiency, using 129×C57Bl/6 (F_2_) *Il9* ^−/−^ mice backcrossed six times onto a BALB/c background, led to modest reductions in mast cell numbers but did not alter the outcome of infection with the flagellated intestinal protozoan parasite *Giardia lamblia* [172]. Moreover, IL-4 has been shown to control intestinal parasitic infections in an IL-5/IL-9/IL-13 triple-knockout mouse [173]. Collectively, these data suggest that the T_H_9 subset may serve as an important adjunct to the T_H_2 response in certain parasitic infections; however, it appears to be superfluous in certain circumstances where T_H_2 responses suffice. In support, IL-9 has been shown to precede and regulate T_H_2-associated cytokine responses in certain parasitic infections [169], [170]. Given that T_H_9 cells have been reported to produce the CCR4 ligands CCL17 and CCL22 [164], and studies that demonstrate that IL-9 can induce expression of the inflammatory chemokine CCL11 by smooth muscle cells [174], [175], early IL-9 responses may be important for amplifying CCR3^+^ eosinophil [176] or CCR3^+^/CCR4^+^ T_H_2 cell responses at sites of microbial infection.

The function of IL-9 in other microbial infections is less well-defined with conflicting conclusions having been reached to date. Following reports that bronchial secretions from infants with respiratory syncitial virus (RSV) bronchiolitis contained high levels of IL-9 [177], the function of IL-9 was specifically investigated in a murine model of RSV vaccination and infection. Antibody-mediated neutralisation of IL-9 in these models resulted in enhanced viral clearance from the lungs and had varied effects on pathology depending on the timing of IL-9 depletion [178]. In contrast to the detrimental roles of IL-9 in the RSV model, prophylactic administration of recombinant IL-9 into mice infected with a lethal dose of *Pseudomonas aeruginosa* enhanced survival via suppression of inflammatory cytokines including IFN-γ and TNF-α, and induction of the immunomodulatory cytokine IL-10 [179]. Endogenous IL-9 induction was detected in spleens of mice infected with sublethal, but not lethal, doses of *P. aeruginosa*; however, the precise cellular source of this IL-9 was not explored [179]. IL-9-secreting CD4^+^ T cells have also been detected in humans with *M. tuberculosis* infection [180]; however, the functional significance of these cells in immunity to this pathogen has yet to be investigated. It is clear that a great deal of work is still required to delineate the function of T_H_9 cells in the context of microbial infections. Owing to the recent discovery of this subset, the majority of studies investigating the function of IL-9 in protective immunity have utilised systemic means of IL-9 blockade/neutralisation such as the use of antibody-mediated neutralisation or IL-9-deficient mice. However, IL-9 can be elicited from multiple cell types including ILCs, mast cells, Tregs, and natural killer T cells [181]. Therefore, more refined studies making use of the recently described INFER mice [169], which will be useful to detect IL-9-expressing cells in real time, IL-9 fate-mapping reporter mice [182], or mice with specific deletions of the T_H_9-specific transcription factor PU.1 in the T cell compartment [164] are required to determine which microbial infections elicit a T_H_9 response and whether this response is protective.

Taken together, it appears that the T_H_9 response may play significant roles in immunity to certain intestinal parasites and contribute to host protection via amplification of the infectious site's chemotactic potential and mediating mast cell and basophil activation. Although less well-understood, the T_H_9 response may also participate in a diverse array of other infections; however, it appears that these cells play a role supplementary to T_H_2 cells and in some cases a potentially detrimental role in host defences.

## T Follicular Helper (T_FH_)

**Figure 3:**
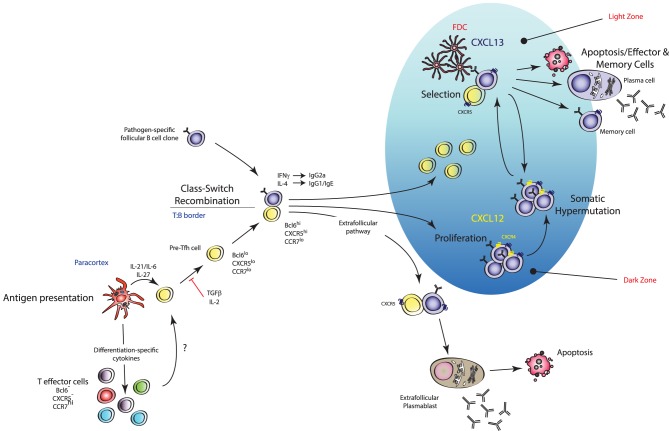
Mechanism of action of T_FH_ cells. T_FH_ cells are effector T_H_ cells that govern the quality and magnitude of an antibody response via regulation of B cell selection, differentiation, proliferation, and class switch recombination. T_FH_ cells execute these effector functions via expression of various cell surface proteins and cytokines (including IL-21). They are generated during antigen presentation in the T cell areas of secondary lymphoid organs in the presence of IL-21 and IL-6, which is thought to upregulate their master transcription factor Bcl6 (pre-T_FH_), after which they migrate to the T∶B border where interaction with cognate B cells regulates a number of processes including promoting survival of recently activated B cells, regulating the fate decision of a B cell down extrafollicular plasmablast or germinal center (GC) B cell differentiation pathways, and induction of class switch recombination in GC B cells. Stable interactions with cognate B cells at this border also consolidate the T_FH_ cell programme (pre-T_FH_ to T_FH_ cell differentiation) with further upregulation of Bcl6 and entry into developing GCs. Within GCs, T_FH_ cells are crucial for the regulation of affinity maturation, development of memory B cell populations, and high-affinity antibody responses via regulation of long-lived plasma cell differentiation.

The T_H_1, T_H_2, T_H_17, T_H_22, and T_H_9 subsets represent populations of effector helper T cells that contribute to immune responses at peripheral sites of infection. However, within secondary lymphoid organs, populations of effector CD4^+^ T cells interact with clonally selected B cells to produce humoral immunity by providing crucial signals that regulate B-cell survival, proliferation, affinity maturation, CSR, and differentiation into memory B– or long-lived plasma cells. This molecular cross-talk between B and T cells occurs in two waves: first at the T-B border where CD4^+^-T-cell-derived cytokines instruct developing B cells to switch to an appropriate isotype and actively form specialised structures known as germinal centres (GC), followed by further interactions within the GC that ultimately determine the quality of antibody response generated [183]. Prior to 2004, CD4^+^ T cells localised to GCs were thought to be a branch of canonically derived T_H_1 or T_H_2 cells that migrated into B cell follicles to coordinate CSR to IgG_2A_ and IgG_1_ via IFN-γ and IL-4, respectively [184]. However, recent data have demonstrated that these GC-localised T cells, now referred to as follicular T_H_ cells (T_FH_), are in fact a distinct differentiation lineage (Figure 1 and Figure 3). These cells are characterised by expression of the lineage-specific master regulator Bcl6 (the transcription factor c-Maf is also crucial to this subset) [185]–[187], as well as the ability to produce a range of cytokines including IL-4, IFN-γ, IL-21, and IL-17A [188]–[195] (Figure 1 and Figure 3). T_FH_ cells are also characterised by high expression of the chemokine receptor CXCR5, which mediates migration into B cell follicles and GCs that are rich in CXCL13 [196], [197].

Current models of T_FH_ cell development describe a “two-wave” theory of differentiation: DC-instructed commitment to the T_FH_ cell lineage (i.e., pre-T_FH_ cell differentiation) followed by B-cell-instructed consolidation of the T_FH_ cell programme (i.e., GC-resident T_FH_ cells) [198]. It is thought that T_FH_ cells selectively differentiate from naïve precursors with the highest affinity to any given antigen [199], consistent with reports that the magnitude of T_FH_ cell generation is dependent on the dose of Ag made available to the T cell during its interaction with a DC [200]. STAT3 activation downstream of IL-6 and/or IL-21 has also been shown to promote early commitment to the T_FH_ cell lineage via induction of their master transcriptional regulator Bcl6 [193], [201], [202]. Interestingly, STAT1 and STAT4 activation downstream of IL-6 and IL-12 signalling respectively, two transcription factors known to promote T_H_1 cell differentiation, have been shown to induce expression of Bcl6 in CD4^+^ T cells [203]–[206]. Nakayamada et al. provided evidence that T_H_1 cell differentiation may occur through a T_FH_-cell-like transitional state where T-bet and Bcl6 are co-expressed in the same cell [207]. T-bet was shown to eventually outcompete Bcl6 function when IL-12 signalling persists leading to downregulation of Bcl6, and T_H_1 lineage fate commitment then predominating [207]. Thus, the earliest events of DC-instructed commitment to the T_FH_ cell lineage are complex and likely involve the integration of numerous microenvironmental signals [208]. Importantly, differentiation of T_FH_ cells appears to be independent of the other known effector subsets as mice deficient in genes critical to T_H_1, T_H_2, and T_H_17 lineage development do not display marked differences in T_FH_ cell induction [193]. Early T_FH_ cell commitment is coupled with the downregulation of CCR7 (the ligands of which are in highest concentration in T cell zones of SLOs) and upregulation of CXCR5 (the ligands of which are in highest concentration in the B cell zones), facilitating the movement of the pre-T_FH_ cell to the T-B border [209]. Stable interactions with cognate B cells at this border are required for the terminal differentiation of the T_FH_ cell programme and are coupled with further upregulation of Bcl6, thus promoting expression of genes required for B cell help, and CXCR5, which facilitates the migration of these cells deeper into the B cell zone and into GCs where they execute their predominant effector function [198], [210].

Given that the ability of the host to generate high-affinity neutralising/opsonising antibody responses via the GC reaction is seminal for defence against a number of ever-evolving pathogens, the function of T_FH_ cells is predominantly associated with regulating the process of antibody affinity maturation through governance of selection, survival, proliferation, and differentiation of high-affinity, pathogen-specific B cell clones. T_FH_ cells execute these effector functions via the wealth of cell-surface and soluble proteins that they express (reviewed in [184]) including CD40L, which interacts with GC-B-cell-expressed CD40 and imprints an anti-apoptotic transcriptome in the responding B cell [211], [212], and IL-21, which promotes the proliferation of GC B cells [190], [213], [214] and drives their differentiation toward the plasma cell compartment in both mice [215] and humans [216]–[218]. Therefore, it is perhaps of no surprise that individuals with mutations in these factors exhibit defects in generating isotype-switched high-affinity antibody responses and, thus, are more prone to opportunistic infection. For instance, patients with mutations in *Cd40lg* develop the primary immunodeficiency hyper-immunoglobulin M syndrome, characterised by a severe deficit in GC development and lack of circulating isotype-switched immunoglobulin, and, subsequently, an increase in susceptibility to recurrent bacterial infections and are unresponsive to vaccination [214], [219], [220]. Similarly, patients with a mutation in the SLAM-associated protein (SAP) encoding gene *Sh2d1a*, a signalling protein expressed by T_FH_ cells critically required for the formation of stable T∶B conjugates [221], [222] and GC-T_FH_ cell expression of IL-4 [223], develop X-linked lymphoproliferative (XLP) disease characterised by an increased susceptibility to a number of pathogens (particularly Epstein-Barr virus infection, which can be fatal in children) due to abortive B cell responses [224].

Central to the ability of the host to generate an effective antibody response to a given pathogen is the decision of B cells to class switch to an appropriate antibody isotype for maximum effector function tailored to the nature of the microbe. CSR to IgG_2A_ is driven by IFN-γ and plays a vital role in the neutralisation of viruses, complement fixation, and opsonisation of microbes via the Fc portion. Conversely, IL-4-mediated CSR to IgG_1_ and IgE is essential for antibody-mediated cell cytotoxicity mechanisms in the context of antiparasitic immunity. Despite the clear demonstration by multiple groups that differentiation of Bcl6^+^ T_FH_ cells *in vivo* is independent of T_H_1, T_H_2, or T_H_17 differentiation pathways [185]–[187], how T_FH_ cells could coordinate CSR during particular infections via IL-4 and/or IFN-γ posed something of a paradox. Using reporter systems and other elegant approaches, recent work has revealed that T_FH_ cells can differentiate in a variety of priming environments and that the cytokine milieu present during T cell activation likely favours the production of IFN-γ or IL-4 by T_FH_ cells in the context of T_H_1– or T_H_2-polarising infections, respectively (Figure 3). Using 4get/KN2 dual reporter mice, which faithfully report cells that have actively transcribed from the *Il4* locus and cells actively producing IL-4 [225], Reinhardt and colleagues demonstrated that during infection with the type-2 pathogens *L. major* or *N. brasilienis*, IL-4 production in draining lymph nodes was restricted to *bona fide* T_FH_ cells that were phenotypically and functionally distinct from canonical T_H_2 cells [226]. T_FH_-cell-derived IL-4 was important for driving parasite-specific IgG_1_ responses as GC B cells sorted from IL-4-producing T_FH_∶GC B cell conjugates were actively undergoing CSR to IgG_1_ [226]. Using IFN-γ reporter mice, the same study demonstrated that GC B cells sorted from IFN-γ-producing T_FH_∶GC B cell conjugates were actively undergoing CSR to IgG_2A_ [226]. Consistent with these observations, Lutjhe and colleagues recently demonstrated that T_FH_ cells favoured the production of IFN-γ following influenza infection [227]. However, development of IgG_2C_^+^ (C57Bl/6 mouse equivalent of IgG_2A_) GC B cells was unperturbed in chimeric mice reconstituted with IFN-γ-deficient CD4^+^ T cells [227]. Collectively, the results of these studies suggest that T_FH_-derived IL-4 is seminal for generation of pathogen-specific IgG_1_ antibody responses whilst T_FH_-derived IFN-γ is not essential for an IgG_2A_ antibody response, although it may play a supplementary role in this process.

Much controversy still exists in the field of T_FH_ cell biology regarding the origin of these cells during pathogen encounter. Although it is apparent that the differentiation programmes of T helper cells with B-cell helper function are separate to other effector T_H_ cell subsets as described above, studies have demonstrated that non-T_FH_ effector T cells can re-differentiate to the T_FH_ lineage [228], [229] (Figure 3). T_FH_ cells have been described to arise from T_H_1 in the context of LCMV infection [230]; from T_H_2 cells in the context of *Heligmosomoides polygyrus*, *S. mansoni*, and *N. brasiliensis* infection [188], [226], [229]; and from T_H_17 cells in the Peyer's patches, which was shown to be critical for antigen-specific IgA responses [231]. From a clinical perspective, the ability of the host to generate high-quality antibody responses governs the success of most currently available vaccination strategies; therefore, understanding the differentiation pathways and cytokine-secreting repertoires of T_FH_ cells under different immunising conditions is imperative to the design of vaccines that generate high-affinity antibody responses with the appropriate dominant antibody isotype tailored to the nature of the microbe of interest. The recent development of cytokine fate-mapping reporter mice and generation of Bcl6 and IL-21 reporter mice that faithfully map T_FH_ cells during an immune response [227], [228] should facilitate the collection of important information regarding these processes.

## Concluding Remarks

The adaptive immune response has a broad array of strategies to combat infectious, potentially pathogenic agents. One of the most important strategies utilised is to tailor the immune response to combat particular classes of microbial agents, and T_H_ cell subsets play a crucial role in this process. Significant progress has dramatically improved our understanding of T_H_ cell biology with a number of new subsets recently being identified and discussed in this review, as well as emerging effector T_H_ cell phenotypes, such as granzyme B-expressing cytolytic CD4^+^ T cells found in certain viral infections [232] and subsets of T_H_ cells dedicated to production of IL-21 (T_H_21) [233], that have not yet been shown to be a distinct effector lineage and are not discussed here but warrant further study. Together, these cells give the adaptive immune response the potential to deliver antigen-specific inflammatory responses that instruct and complement pathogen-tailored innate inflammatory responses. These T_H_ responses differentially combat extracellular pathogens, enhance cell-mediated immunity required to combat intracellular pathogens, and promote humoral immunity to produce antibodies that target pathogens. Future challenges include further dissection of this system to identify other potentially important subsets and identifying ways in which to utilise this knowledge to develop better strategies to combat infectious pathogens. Ascertaining such knowledge will be crucial for determining whether future vaccination strategies: i) elicit robust T_FH_ cell responses with the appropriate cytokine-secreting repertoire to induce an antibody response tailored to the nature of the pathogen; and ii) activate the appropriate components of the innate immune system that induce a priming microenvironment driving differentiation of the desired pathogen specific-effector T_H_ cell subset and/or -CTL activation such that upon contact with the live pathogen, all aspects of the adaptive immune system are armed to promote effective, pathogen-tailored clearance of the infectious agent. Thus, increased understanding of the complex dynamics of T_H_ differentiation in the context of microbial infections should lead to improved vaccine efficacy for a wide range of human pathogens.
